# Recurrent Neural Network Based Link Quality Prediction for Fluctuating Low Power Wireless Links

**DOI:** 10.3390/s22031212

**Published:** 2022-02-05

**Authors:** Ming Xu, Wei Liu, Jinwei Xu, Yu Xia, Jing Mao, Cheng Xu, Shunren Hu, Daqing Huang

**Affiliations:** 1 College of Electronic and Information Engineering, Nanjing University of Aeronautics and Astronautics, Nanjing 210016, China; xuming1804@nuaa.edu.cn (M.X.); xiayu2020@nuaa.edu.cn (Y.X.); xcheng@nuaa.edu.cn (C.X.); radiouav@nuaa.edu.cn (D.H.); 2 School of Electrical and Electronic Engineering, Chongqing University of Technology, Chongqing 400054, China; xujinwei@2018.cqut.edu.cn (J.X.); maojing@cqut.edu.cn (J.M.); hsr71@cqut.edu.cn (S.H.)

**Keywords:** low power wireless links, link quality prediction, recurrent neural network, link quality indicator, time series, temporal correlation

## Abstract

One of the main methods for link quality prediction is to predict the physical layer parameters first, and then evaluate the link quality based on the mapping models between such parameters and packet reception ratio (PRR). However, existing methods often ignore the temporal correlations of physical layer parameter series and rarely consider the influence of link fluctuations, which lead to more errors under moderate and sudden changed links with larger fluctuations. In view of these problems, this paper proposes a more effective link quality prediction method RNN-LQI, which adopts Recurrent Neural Network (RNN) to predict the Link Quality Indicator (LQI) series, and then evaluates the link quality according to the fitting model of LQI and PRR. This method accurately mines the inner relationship among LQI series with the help of short-term memory characteristics of RNN and effectively deals with link fluctuations by taking advantage of the higher resolution of LQI in the transitional region. Compared with similar methods, RNN-LQI proves to be better under different link qualities. Especially under moderate and sudden changed links with larger fluctuations, the prediction error reduces at least by 14.51% and 13.37%, respectively. Therefore, the proposed method is more suitable for low power wireless links with more fluctuations.

## 1. Introduction

Low power wireless systems usually employ radio transceivers with low transmit power to reduce the energy consumption of communication. These systems such as wireless sensor network have been successfully used in many fields, including object tracking, habitat monitoring, industrial control and so on [1,2]. Low transmit power generally means low link margin, which makes the link quality prone to fluctuate when channel environment changes. Therefore, stability of these low power links is poor. In order to assist upper layer protocols to select better links, the adverse effects of link fluctuations must be considered when designing link quality prediction (LQP) methods. If the trend of link changes could be perceived in advance, link fluctuations of low power wireless communications would be handled more effectively.

Packet reception ratio (PRR) is the most direct metric to describe link quality. However, existing studies have proven that the agility of directly using PRR for link quality prediction is very poor [3]. It means that long-term statistics are needed to obtain reasonable PRR estimations, which inevitably affects the timely response to link fluctuations. This problem can be solved effectively by employing physical layer parameters such as Received Signal Strength Indicator (RSSI), Link Quality Indicator (LQI), and Signal-to-Noise Ratio (SNR) for link quality prediction. Physical layer parameters are more agile than PRR, which means that small time windows are adequate for such parameters to describe link quality accurately.

By directly predicting physical layer parameters computed within small time windows and then evaluating link quality according to the mapping models between such parameters and PRR, the agility could be effectively improved without sacrificing the accuracy of prediction. However, existing methods often ignore the temporal correlations of physical layer parameter series when predicting. Thus, the inner relationship among series cannot be mined accurately, which leads to large deviations between the predicted values and the actual ones. Meanwhile, they rarely consider the influence of link fluctuations, resulting in higher errors under moderate and sudden changed links with larger fluctuations. Therefore, it is difficult to meet the requirements of low power wireless link quality prediction.

This paper proposes a more effective link quality prediction method RNN-LQI, which adopts Recurrent Neural Network (RNN) to predict LQI counted within small time windows, and then evaluates link quality according to the fitting model of LQI and PRR. The advantages of this method are mainly two-fold: Firstly, it makes use of the short-term memory characteristics of RNN to accurately mine the inner relationship among LQI series. Secondly, it takes advantage of the higher resolution of LQI in the transitional region to effectively handle link fluctuations. To analyze the performance of RNN-LQI, real link traces are collected and used to train and validate the proposed method. Meanwhile, typical methods with similar structures are chosen for comparison.

Experimental results show that RNN-LQI is more accurate under different link qualities. Especially under moderate and sudden changed links with more fluctuations, the prediction error reduces at least by 14.51% and 13.37%, respectively. It means that the proposed method is more suitable for low power wireless links with more fluctuations. Major contributions of this paper are summarized as follows: (1) two main sources of error for the link quality prediction based on physical layer parameter prediction and mapping are identified; (2) a more effective link quality prediction method is proposed, which could suppress both error sources of prediction and mapping simultaneously; (3) with real link traces collected, the proposed method is proved to be superior for low power wireless links with more fluctuations.

The rest of this paper is organized as follows: Related works are given in Section 2. This is followed by the design motivation in Section 3. The proposed algorithm is described in detail in Section 4. With the experimental setup in Section 5, performance comparison with similar approaches is discussed in Section 6. Finally, conclusions are presented and suggestions are made for future works.

## 2. Related Works

Wireless link quality prediction is essential for low power wireless networks. Different parameters could be used for link quality prediction, such as parameters directly acquired from transceivers (usually referred to as hardware metrics). Baccour et al. [3] divided existing methods into three categories according to the parameters they used, which are hardware metric-based methods, software metric-based methods, and hybrid metric-based methods.

PRR is the most common software metric for link quality prediction. Woo et al. [4] found that PRRs counted within fixed time windows are not stable and proposed to use exponentially weighted moving average filter to obtain more stable estimations. Liu et al. [5] pointed out that existing methods cannot self-adapt to link fluctuations and proposed a fluctuation adaptive link quality estimator, which dynamically adjusts the smoothing factor according to the degree of link fluctuations. These efforts have indeed improved the accuracy and stability of link quality prediction. However, the disadvantage that large time windows must be used to obtain accurate PRR estimations still exists. That is to say, the agility of directly using PRR is still poor.

Physical layer parameters such as RSSI, SNR and LQI could be easily acquired from transceivers. These parameters reflect the wireless signal quality most directly. Due to their high correlations with PRR, they have been extensively used for link quality prediction. Compared with PRR, the most obvious advantage of these parameters is that they are more agile for link quality prediction. Xu et al. [6] pointed out that average RSSI and LQI computed with only 10 packets are adequate to reflect the link quality accurately, while 50 packets have to be used to achieve similar effects when using PRR.

Directly using physical layer parameters to evaluate link quality qualitatively is a common practice in early studies based on hardware metrics. Srinivasan et al. [7] pointed out that whether a link is good or not could be determined quickly and accurately using RSSI. Variance of LQI is a good indicator to distinguish the quality of links and good effects could be achieved only using 10 packets [8]. To utilize the information carried by different physical layer parameters effectively, Boano et al. [9] proposed to fuse SNR and LQI to construct a new metric Triangle. It is shown that this new metric is superior for classifying the link quality. Aiming at the problem that the weight of LQI is too high in Triangle, Liu et al. [10] proposed to use weighted Euclidean distance for fusion to make full use of the information contained in SNR and LQI.

Quantitative prediction of link quality could be realized by introducing mapping models between physical layer parameters and PRR. Ye et al. [11] proposed to predict the link quality with the mapping model of RSSI and PRR, in which the model is established using logistic regression. Similarly, Senel et al. [12] proposed to predict the link quality according to the mapping model of SNR and PRR. Gomez et al. [13] established a piecewise linear model of LQI and PRR, which is used to predict the link quality. The link quality predictor 4C proposed by Liu et al. [14] trains the historical data of physical layer parameters and PRR with different algorithms. Considering that 4C needs offline model training, the authors proposed a real-time predictor TALENT, which implements online model training using stochastic gradient descent learning algorithm [15]. Xue et al. [16] proposed to predict the probability-guaranteed interval boundary of SNR based on random vector function chain and realize quantitative link quality prediction by establishing a mapping model between SNR and PRR.

In recent years, more powerful machine learning algorithms like neural network have been adopted in link quality prediction. The link quality predictor WNN-LQE proposed by Sun et al. [17] employs wavelet neural network (WNN) to predict SNR, and then quantize the link quality through the mapping model of SNR and PRR. In our previous work, RNN was used to predict SNR in order to mine the internal relationship among SNR series more accurately [18]. Liu et al. [19] used WNN to predict LQI, and then quantized the link quality through the mapping model of LQI and PRR. The authors found that their predictor is superior in accuracy to WNN-LQE when there are larger link fluctuations. The reason was attributed to the fact that LQI has higher resolution than SNR in the transitional region.

Physical layer parameters are generally extracted from successfully received packets. Thus, the methods that only use physical layer parameters lose the information relating to unreceived packets. Therefore, the link quality cannot be fully characterized. In comparison, PRR statistics consider the packets not received. That’s why some studies combine software and hardware metrics to predict link quality. The link quality estimator Four-Bit proposed by Fonseca et al. [20] combines physical layer, link layer, and network layer information. It is shown that Four-Bit improves the performance of link quality evaluation remarkably. Baccour et al. [21] proposed F-LQE, in which fuzzy logic is used to fuse four link parameters, including the mean value and variation coefficient of PRR, the link asymmetry, and the mean value of SNR.

Too stable is one obvious disadvantage of F-LQE. Therefore, the link parameters used by fuzzy logic were adjusted so as to realize higher agility and accuracy [22,23]. Rekik et al. [22] proposed Opt-FLQE, in which the link parameters are changed to the mean value of PRR, the link asymmetry, the number of retransmissions of transmitter, and the mean value of SNR. Similarly, the ELQET proposed by Jayasri et al. [23] utilizes another four link parameters, which are the PRR mapped from LQI, the Kalman filtered SNR, the variation coefficient of PRR and the mean value of LQI. Although these methods look better than F-LQE, the introduction of software metrics inevitably affects their agility, which makes them unsuitable for links with more fluctuations.

## 3. Design Motivation

From the descriptions of Section 2, it can be found that one of the main methods for link quality prediction is to predict the physical layer parameters first, and then evaluate the link quality based on the mapping models between such parameters and PRR. There are two main sources of error for this type of method, one is the prediction error of physical layer parameters, and the other is the mapping error from physical layer parameters to PRR. In order to improve the accuracy of such methods, it is necessary to reduce both errors at the same time. However, existing methods fail to solve this problem well. On the one hand, the temporal correlations of physical layer parameter series are often ignored when predicting. Thus, the inner relationship among series cannot be mined accurately, which leads to large deviations between the predicted values and the actual ones. On the other hand, the influence of link fluctuations is not considered sufficiently, resulting in higher errors under moderate and sudden changed links with larger fluctuations. Therefore, it is difficult to meet the requirements of low-power wireless link quality prediction. Considering the obvious temporal order of physical layer parameter series, it would be feasible to realize more accurate prediction by making full use of these temporal correlations.

**Figure 1:**
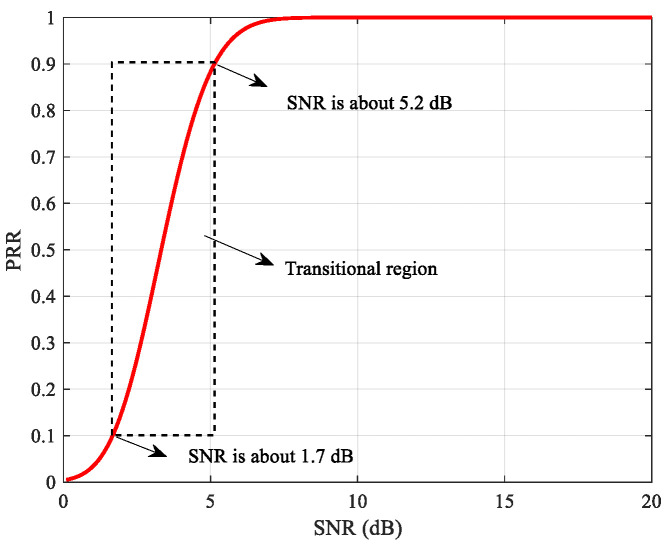
Theoretical Model of SNR and PRR.

**Figure 2:**
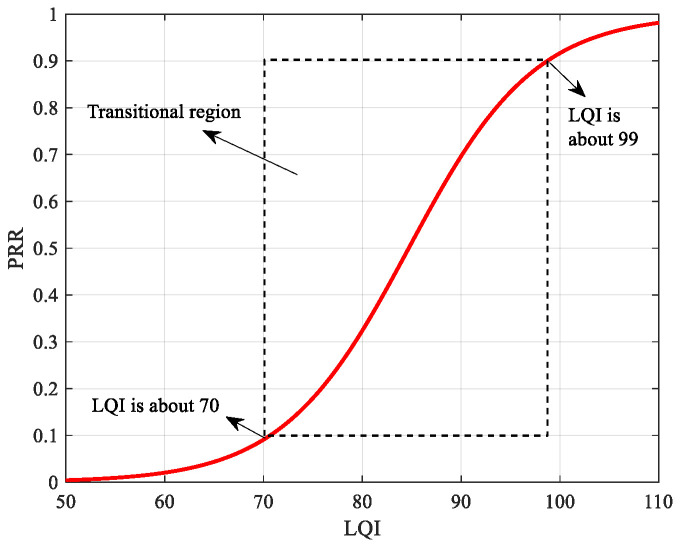
Fitting Model of LQI and PRR.

Figure 1 and Figure 2 demonstrate the relationship between typical physical layer parameters and PRR. Given the similarity between RSSI-PRR relationship and SNR-PRR relationship, only the latter one is given here as an example. The relationship between SNR and PRR is usually described with the theoretical model [24], while the relationship between LQI and PRR is generally fitted by logistic regression or hyperbolic tangent model using the measured data from specific environments [14,15,19]. It can be seen that the SNR~PRR model is much steeper in the transitional region, which means that slight link fluctuations will lead to larger PRR differences. In contrast, the LQI~PRR model has much higher resolution in the transitional region. For every 10% change of PRR in the transitional region, LQI changes by about 3.625, while SNR only changes by about 0.4375 dB. Therefore, if choosing higher-resolution physical layer parameters, the impacts of their fluctuations on the mapped PRR may be reduced, thus improving the adaptability to fluctuations of moderate links and sudden changed links.

**Figure 3:**
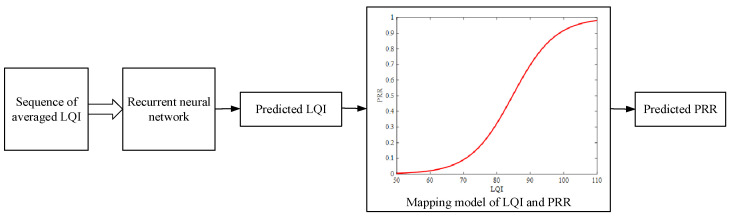
Structure of the proposed RNN-LQI.

Based on the above analysis, this paper proposes a more effective link quality prediction method RNN-LQI, which adopts RNN to predict the LQI series, and then evaluates the link quality according to the fitting model of LQI and PRR, as shown in Figure 3. On the one hand, this method could better mine the inner relationship among LQI series with the help of the short-term memory capability of RNN, which helps to reduce the impacts of prediction errors. On the other hand, it could alleviate the impacts of link fluctuation on PRR mapping by taking advantage of the higher resolution of LQI in the transitional region, which helps to reduce the impacts of mapping errors. Therefore, RNN-LQI could suppress both error sources of prediction and mapping simultaneously, which results in more accurate link quality prediction.

## 4. RNN-LQI Implementation

### 4.1. Recurrent Neural Network

**Figure 4:**
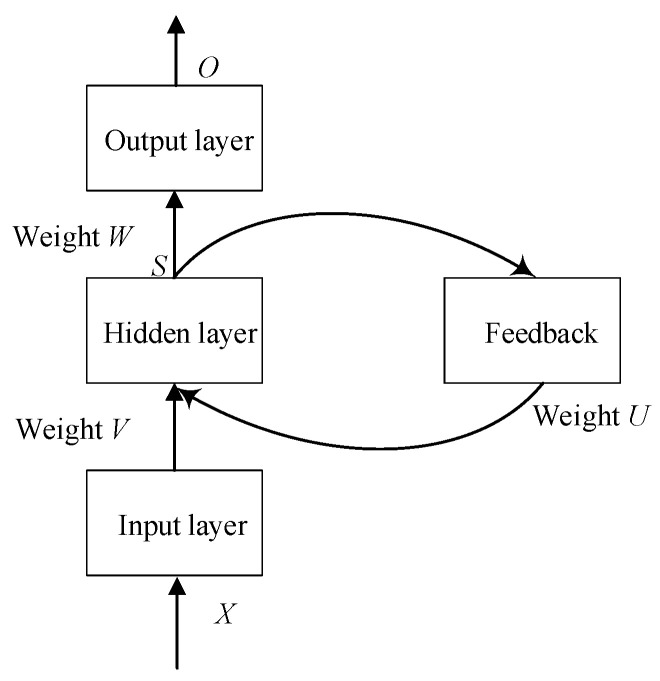
Structure of RNN.

Recurrent neural network possesses short-term memory capability because output information of the past moments is internally feedbacked, stored and utilized. Different from the feedforward neural networks, a ring structure is introduced in the hidden layer of RNN, as shown in Figure 4. This structure makes the output state of RNN at current moment determined by the input state at that moment and the output state at previous moment simultaneously. That is to say, RNN is able to deal with dynamic changes in a short duration. Therefore, RNN is quite effective for data with sequential characteristics and able to effectively mine the hidden temporal information of data. Given its effectiveness, RNN has been widely used in many fields of wireless network research [25].

Execution of RNN includes two phases: the first phase is for forward data propagation and the second phase is for backward error propagation. Denote the input vector of input layer as *X*, and the output vectors of hidden layer and output layer as *S* and *O*, respectively. Meanwhile, set the weight parameter matrix from the input layer to hidden layer as *V* and the weight parameter matrix from the hidden layer to output layer as *W*, respectively. Similarly, *U* presents the parameter matrix of the ring structure in the hidden layer. Defining the number of neurons in the input layer, hidden layer, and output layer is *n*, *m* and *o*, respectively. In the first phase, the input and output of the *j*-th hidden layer neuron could be expressed as follows [26]:

(1) $${net}_{j}(t)=\sum_{i=1}^{n}x_{i}(t)v_{ij}+\sum_{l=1}^{m}h_{l}(t-1)u_{jl}$$

(2) $$h_{j}(t)=f(net_{j}(t))$$

where *v_ij_* denotes the connection weight from the *i*-th input layer neuron to the *j*-th hidden layer neuron, *u_jl_* denotes the connection weight from the current *j*-th neuron to the previous *l*-th neuron in the hidden layer. This is determined by the ring structure introduced, which makes the output state at current moment also relate to the output state at previous moment. *f* denotes the transfer function of hidden layer, for which Sigmoid function was used in this paper.

Input and output of the *k*-th neuron in the output layer could be computed as follows:

(3) $$net_{k}(t)=\sum_{j=1}^{m}h_{j}(t)\omega_{jk}$$

(4) $$y_{k}(t)=f(net_{k}(t))$$

where *ω_jk_* represents the connection weight from the *j*-th neuron in the hidden layer to the *k*-th neuron in the output layer.

Then, the objective error function of RNN could be computed as follows:

(5) $$E=\frac{1}{2}\sum_{k=1}^{o}{\left(o_{k}-y_{k}\right)}^{2}$$

where *o_k_* represents the actual output.

In the backward error propagation phase, parameters of RNN are updated based on the gradient descent strategy. Learning rate *η* (*η* > 0) is defined for *E*. Meanwhile, momentum factor *λ* (0 < *λ* < 1) is designed to accelerate the learning speed and avoid appearance of local optimum. Thus, the updating expressions of the connection weights are as follows:

(6) $$\omega_{jk}(t+1)=\omega_{jk}(t)-\eta \frac{\partial E}{\partial \omega_{jk}}+\lambda [\omega_{jk}(t)-\omega_{jk}(t-1)]$$

(7) $$v_{ij}(t+1)=v_{ij}(t)-\eta \frac{\partial E}{\partial v_{ij}}+\lambda [v_{ij}(t)-v_{ij}(t-1)]$$

(8) $$u_{jl}(t+1)=u_{jl}(t)-\frac{\partial E}{\partial u_{jl}}+\lambda [u_{jl}(t)-u_{jl}(t-1)]$$

where

(9) $$\frac{\partial E}{\partial \omega_{jk}}=-(o_{k}-y_{k})y_{k}(1-y_{k})h_{j}$$

(10) $$\frac{\partial E}{\partial v_{ij}}=-\sum_{k=1}^{o}(o_{k}-y_{k})y_{k}(1-y_{k})\omega_{jk}h\prime_{j}x_{i}$$

(11) $$\frac{\partial E}{\partial u_{jl}}=-\sum_{k=1}^{o}\left(o_{k}-y_{k})y_{k}(1-y_{k})\omega_{jk}h\prime_{j}h_{l}(t-1\right)$$

### 4.2. RNN Based LQI Prediction

As shown in Figure 3, the first stage of RNN-LQI is a RNN based LQI predictor. In this paper, the number of neurons in the input layer of RNN is set as *n* = 10 and the number of neurons in the output layer of RNN as *o* = 1. In other words, a history series of 10 LQIs constitutes the input vector, and the predicted LQI of the next time window constitutes the output vector. The number of neurons in the hidden layer of RNN could be determined as follows:

(12) $$m=\sqrt{n\times o+1.6799\times n+0.9298}\approx 5$$

**Figure 5:**
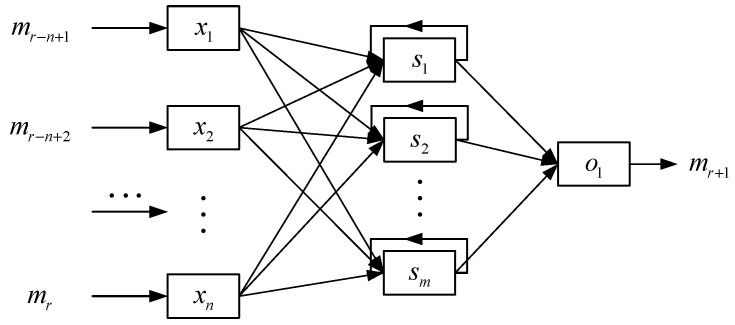
Structure of the LQI Predictor based on RNN.

Assuming that LQI values of the target wireless link in a period of time are expressed as *L*(*N*) *=* {*l*_1_, *l*_2_, ..., *l_N_*}, then the series of averaged LQI could be calculated by window averaging and expressed as *M*(*A*) *=* {*m*_1_, *m*_2_, *…*, *m*_A_}, where *A =* [*N*/*W*] denotes rounding of *N*/*W* and *W* is the size of time window. More specifically, the averaged LQI is calculated as follows:

(13) $$m_{k}=\frac{l_{(k-1)W+1}+l_{(k-1)W+2}+,\ldots ,+l_{kW}}{W}$$

where *k =* 1, 2, …, *A*. In this paper, *W* is set to 10, i.e., summarizing LQI with small time windows. For the current time window *r*, the input of RNN is the series of averaged LQI with a length of *n*, *m*(*r*) *=* {*m_r_*_−*n*+1_, *m_r_*_−*n*+2_, *…*, *m_r_*}. The output is *m_r_*_+1_, that is, the predicted LQI of the next time window, as shown in Figure 5.

### 4.3. Mapping LQI to PRR

For the convenience of subsequent comparison, this paper adopts the hyperbolic tangent function given in [19] to fit the LQI-PRR mapping model, as shown in (14):

(14) PRR=0.5+0.5×tanh(a×LQI+b)

Using the measured data, coefficients *a* and *b* of the model are obtained by means of least square method, resulting in the LQI-PRR mapping model shown in (15).

(15) PRR=0.5+0.5×tanh(0.0783×LQI−6.6315)

## 5. Experimental Setup

The data used for model training and verification was acquired using two sensor nodes, one used as transmitter and the other used as receiver. The type of these sensor nodes is TelosB, which employs an IEEE 802.15.4 compatible transceiver operating in the 2.4 GHz band [27]. The test was conducted in an outdoor playground of Chongqing University of Technology, which consists of a football field and a track. During the test, nearly no interference existed, and the noise level was about—96.37 dBm. Antenna height of the nodes is 1.2 m. In order to get sufficient link data, we changed the distance between the nodes to simulate links with different qualities, which was implemented by fixing the receiver and moving the transmitter. The initial distance was 5 m and the step size was 1 m. Position of the transmitter was changed until the maximum distance of 115 m was reached.

When the distance between the nodes increases, the received signal becomes weak and more packets prone to be damaged and dropped. That is to say, different link qualities could be simulated in this way. During the test, the transmit power was set to 0 dBm, and the 26th channel was used for communication. Meanwhile, the inter packet interval and packet length were set to 125 ms and 17 bytes, respectively. PRR was computed by the number of packets received successfully, and LQI of these successfully received packets was also recorded.

About 2.67 million data packets were received and recorded from the test lasted for up to 47 h. Four kinds of links were categorized according to their qualities. They are good, moderate, bad, and sudden changed links, respectively, which are typical links for evaluating the performance of link quality predictors [4,12]. Links with PRR higher than 80% are called good links. Such links are very stable and there are almost no fluctuations. Links with PRR smaller than 20% are called bad links. Quality of such links is very poor, and small fluctuations exist. Quality of moderate links lies between those of good and bad links. Meanwhile, larger and more frequent fluctuations exist in moderate links. In other words, moderate links are unstable. Sudden changed links refer to cases in which links change from bad to good or vice versa.

These links were divided into two parts: one part is for model training and the other part is for model verification. Matlab R2018a running on a desktop with Intel Core i7 processor was used for model training and verification. Meanwhile, the trained model was also implemented on sensor nodes for evaluating its execution overhead on platforms with low computation capability. 

## 6. Performance Comparison and Analysis

### 6.1. Candidate Methods for Comparison

The prediction methods proposed in [17,18,19] were chosen to compare with the proposed one in this paper. Main reasons for choosing these three methods for comparison are as follows. Firstly, these methods are of the same type. They all predict physical layer parameters first, and then evaluate the link quality based on the mapping models between such parameters and PRR. Secondly, these methods all adopt neural network to predict physical layer parameters, which could guarantee the fairness of comparison. Thirdly, these methods use different combinations of prediction methods and physical layer parameters. Through comparison, the performance improvement brought by the method proposed in this paper could be directly reflected.

WNN is adopted in [19] to predict LQI of the next moment. Then, quantitative prediction of link quality is realized through the LQI-PRR mapping model. The disadvantage of WNN in this scenario is the lack of memory effect. By comparing with it, the performance improvement benefited from considering the temporal correlations of physical layer parameter series could be directly shown. For fairness and consistency, the mapping model of LQI and PRR was fitted using the method given in [19], as shown in Section 4.3.

RNN is adopted in [18] to predict SNR of the next moment. Then, quantitative prediction of link quality is realized through the SNR-PRR mapping model. This method does not consider the resolution when choosing physical layer parameters. By comparing with it, the impacts of link fluctuations on PRR mapping could be directly reflected. Mapping from SNR to PRR utilizes the theoretical model between them, which is shown as follows [24]:

(16) $$PRR=f(SNR)={\left(1-Q(\sqrt{3.072\times 10^{SNR/10}})\right)}^{l}$$

where *Q*(·) represents the Q-function, *l* is the packet length in bits. It can be seen from Section 5 that the value of *l* is 136.

WNN is adopted in [17] to predict SNR of the next moment. Then, quantitative prediction of link quality is realized through the SNR-PRR mapping model. This method considers neither the temporal correlations of physical layer parameter series nor the resolution of physical layer parameters. Through comparison, the impacts of these two factors on the accuracy of prediction could be comprehensively evaluated. Mapping from SNR to PRR also utilizes the theoretical model between them.

For the convenience of presentation, these methods will be expressed with the combination of prediction methods and physical layer parameters in the following. The methods proposed in this paper, [17,18,19] are expressed as RNN-LQI, WNN-LQI, RNN-SNR and WNN-SNR, respectively. In the comparison, the same data set was used for training and validation.

In order to evaluate the accuracy of link quality prediction quantitatively, root mean squared error (RMSE) was employed. Smaller RMSE means higher prediction accuracy, and vice versa. The calculation of RMSE is shown as follow:

(17) $$RMSE=\sqrt{\frac{1}{t}\sum_{i=1}^{t}{\left(y_{i}-{\hat{y}}_{i}\right)}^{2}}$$

where *y_i_* represents the measured value, *ŷ_i_* represents the predicted one, and *t* represents the number of samples.

### 6.2. Comparative Analysis of LQI Prediction

**Figure 6:**
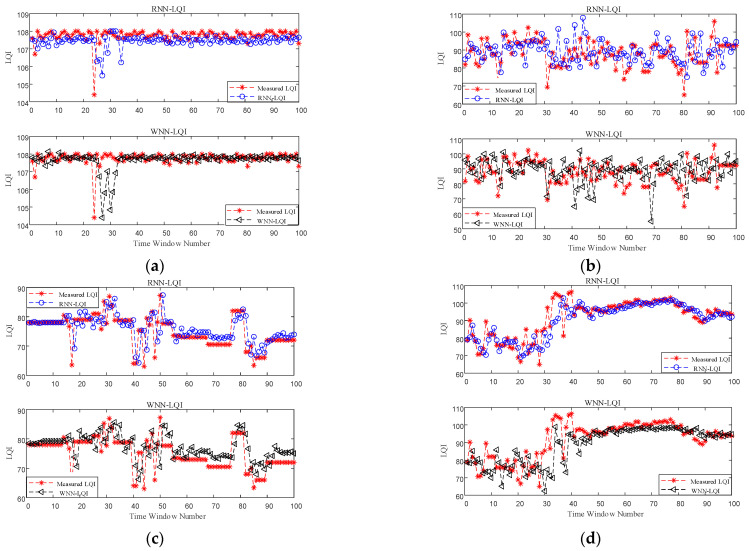
Predicted LQI vs. measured LQI. (**a**) good links, (**b**) moderate links, (**c**) bad links, (**d**) sudden changed links.

Firstly, the performance improvement benefited from considering the temporal correlation of LQI series was analyzed. Only the accuracy of RNN-LQI and WNN-LQI in predicting LQI was compared, and no mapping to PRR was conducted. Figure 6 shows the predicted LQI and measured one for these two methods under different link qualities, in which the Time Window Number denotes the number of time windows used to computing the mean value of LQI. It is obvious that under good and bad links, prediction effects of these two methods are both very good and the predicted LQIs of RNN-LQI are closer to the measured ones. Under moderate links, the predicted LQI of WNN-LQI fluctuates greatly, and the error is as high as 22. In comparison, the predicted LQI of RNN-LQI is closer to the measured one. Under sudden changed links, although WNN-LQI is able to keep up with the sudden change faster, its prediction is not accurate after the link becomes stable. In contrast, the prediction of RNN-LQI is more consistent with actual fluctuations. Combining Figure 6a–d, it can be found that the proposed RNN-LQI is superior to WNN-LQI under different link qualities, which could be attributed to the fact that RNN considers the temporal correlation of LQI series.

Table 1 shows the RMSEs of LQI prediction for RNN-LQI and WNN-LQI under different link qualities. It is obvious that the prediction errors of RNN-LQI under different link qualities are all lower than those of WNN-LQI. Especially under moderate and sudden changed links, RNN-LQI is obviously superior to WNN-LQI. Compared with WNN-LQI, prediction errors of RNN-LQI under moderate and sudden changed links reduce by 17.77% and 28.44%, respectively. That is to say, compared with WNN-LQI, RNN-LQI shows better accuracy in predicting LQI. In other words, when predicting physical layer parameters, it is necessary to consider their temporal correlation.

### 6.3. Comparative Analysis of PRR Prediction

**Figure 7:**
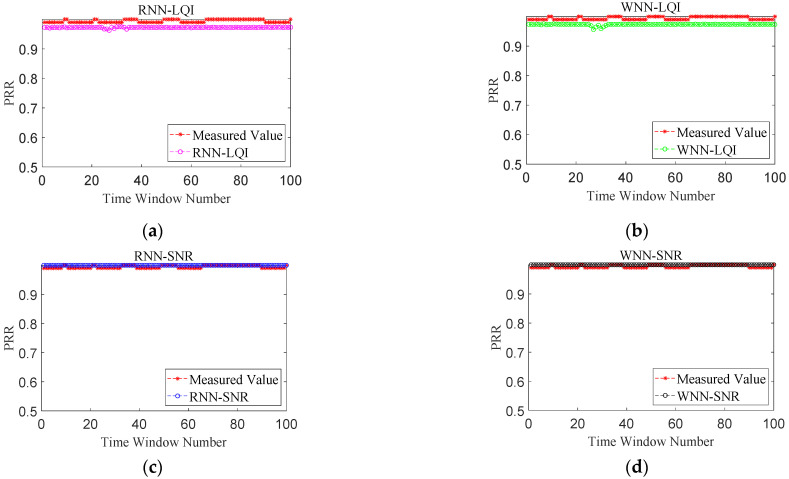
Performance comparison under good links. (**a**) RNN-LQI, (**b**) WNN-LQI, (**c**) RNN-SNR, (**d**) WNN-SNR.

**Figure 8:**
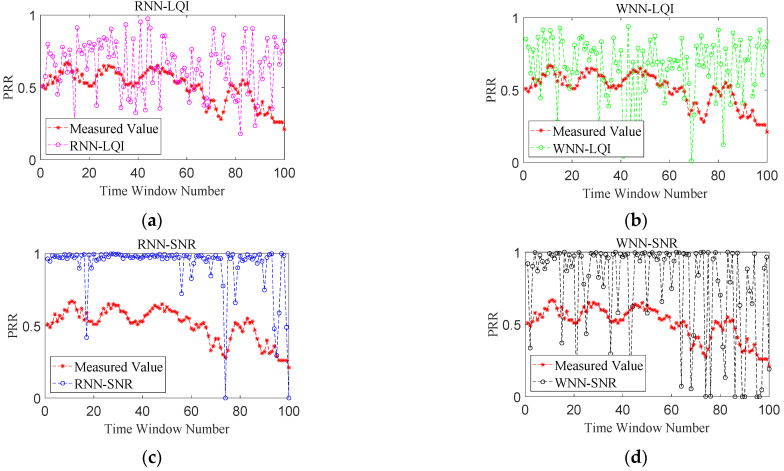
Performance comparison under moderate links. (**a**) RNN-LQI, (**b**) WNN-LQI, (**c**) RNN-SNR, (**d**) WNN-SNR.

**Figure 9:**
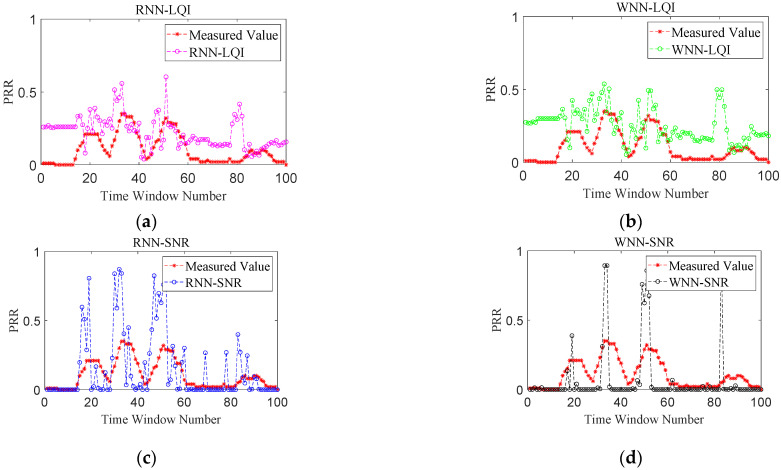
Performance comparison under bad links. (**a**) RNN-LQI, (**b**) WNN-LQI, (**c**) RNN-SNR, (**d**) WNN-SNR.

**Figure 10:**
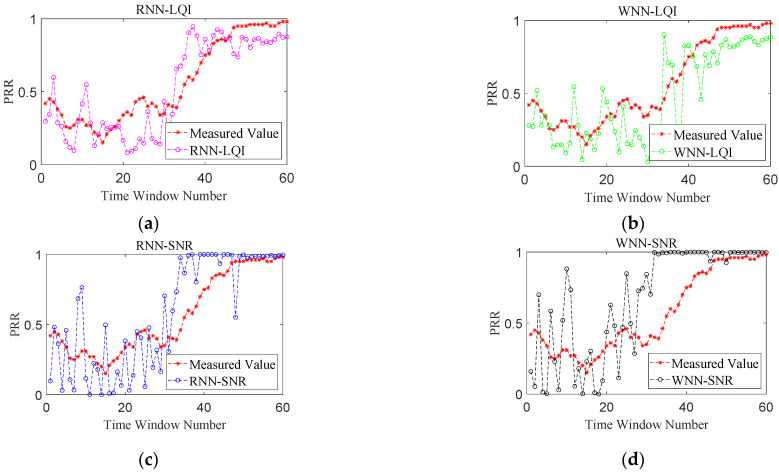
Performance comparison under sudden changed links. (**a**) RNN-LQI, (**b**) WNN-LQI, (**c**) RNN-SNR, (**d**) WNN-SNR.

Figure 7, Figure 8, Figure 9 and Figure 10 show the effects of PRR prediction for all the methods under different link qualities. It is obvious that the prediction effects of all four methods are very good under good links. Among them, the predicted PRRs of RNN-SNR and WNN-SNR with SNR as their target physical layer parameter are closer to the measured values. Under moderate links, the prediction effects of RNN-LQI and WNN-LQI with LQI as the target physical layer parameter are significantly better than RNN-SNR and WNN-SNR. The prediction effects of RNN-SNR and WNN-SNR evidently get worse, with more fluctuations and more predicted PRRs close to 1. RNN-LQI is better than the other three methods under bad links, which is followed by WNN-SNR. The predicted PRRs of RNN-LQI and WNN-LQI with LQI as their target physical layer parameter are closer to the measured values under sudden changed links. Meanwhile, RNN-LQI with memory capability is better.

Table 2 shows the RMSEs of PRR for these four methods under different link qualities. It is obvious that under good links, RNN-SNR and WNN-SNR have the lowest RMSE. Compared to RNN-SNR and WNN-SNR with SNR as their target physical layer parameter, RNN-LQI and WNN-LQI with LQI as their target physical layer parameter have lower RMSEs under moderate links and sudden changed links. Under moderate, bad, and sudden changed links, the prediction errors of RNN-LQI are all smaller than the other three methods. In particular, the prediction errors of RNN-LQI compared with WNN-LQI under moderate, bad, and sudden changed links reduce by 14.51%, 14.43% and 13.37%, respectively. Compared with RNN-SNR and WNN-SNR, the prediction errors of RNN-LQI under moderate, bad, and sudden changed links reduce at least by 37.56%, 12.89% and 27.59%, respectively.

**Figure 11:**
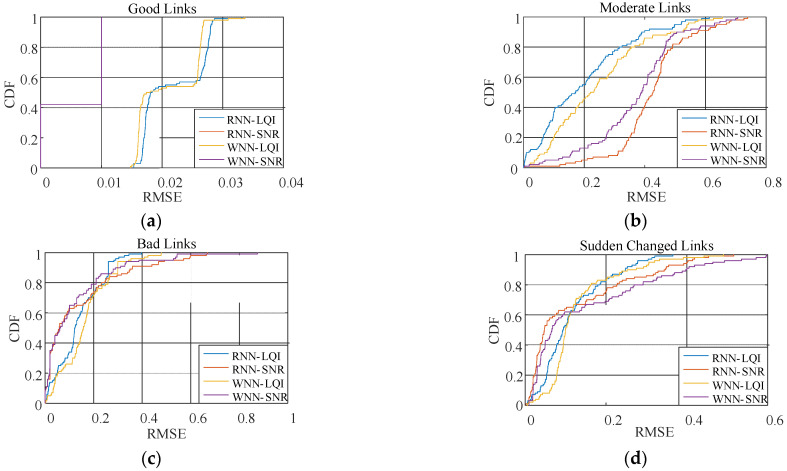
CDF of the RMSE for PRR prediction under different link qualities. (**a**) good links, (**b**) moderate links, (**c**) bad links, (**d**) sudden changed links.

Figure 11 shows the cumulative distribution function (CDF) of the RMSE for PRR prediction under different link qualities. It is obvious that under good links, the methods using SNR look superior to those using LQI. However, RMSEs of all the methods are very low, no more than 0.03. That is to say, the prediction effects of all the methods are very good under good links. Under moderate links, CDF of the RMSE for RNN-LQI lies to the left of the other methods. In other words, RNN-LQI is more accurate. Under bad links and sudden changed links, the cumulative probability of RNN-LQI converges to 1 the fastest, which contributes to the smallest RMSE of RNN-LQI in these links. In brief, RNN-LQI is able to predict the link quality more accurate and reliable than other similar methods.

Combined with the analysis of Section 6.2, it can be seen that compared with WNN-LQI, RNN-LQI is able to predict LQI more accurately. Since the two methods use the same physical layer parameter and mapping model, the performance improvement of RNN-LQI mainly comes from the consideration of temporal correlation of LQI series. That is to say, LQI prediction error is reduced by accurately mining the inner relationship among LQI series. Compared with RNN-SNR, as the two methods use the same prediction method, the performance improvement of RNN-LQI mainly comes from adopting the physical layer parameter with higher resolution in the transitional region. That is to say, the adaptability to link fluctuations is improved by reducing the impacts of physical layer parameter fluctuations on the mapped PRR. Therefore, considering the temporal correlation of physical layer parameter series and the resolution of physical layer parameter mapping to PRR will help to improve the accuracy of link quality prediction. It means that the proposed method is more suitable for low-power wireless links with more fluctuations.

### 6.4. Analysis of Algorithm Convergence and Execution Overhead

According to Liu et al. [14], model training with more computational overhead could be executed on more powerful desktops offline using the test data, while only the trained model needs to be deployed online as it has less computational overhead. This approach is feasible for low power wireless nodes. This section analyzes the convergence when training the model on desktop firstly and then shows the execution overhead when deployed the trained model on sensor nodes.

**Figure 12:**
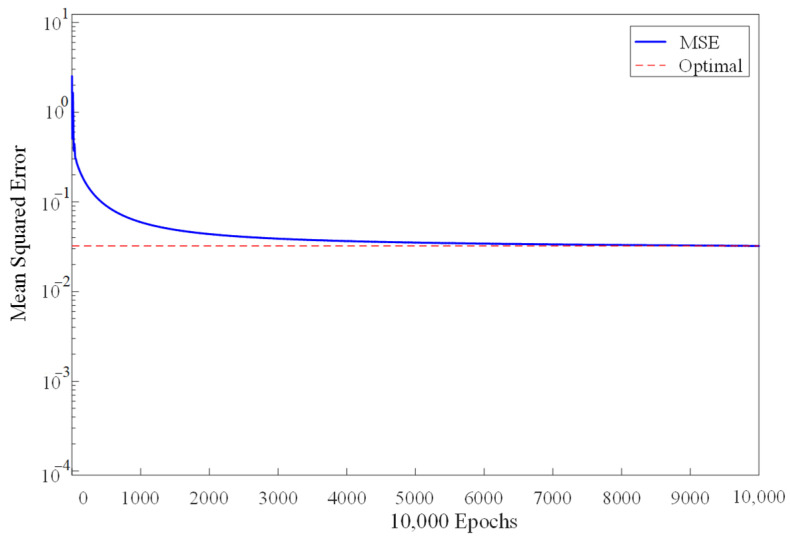
Convergence when training the model on desktop.

Figure 12 shows the convergence when training the model on desktop. It can be seen that with the first 2000 samples, the mean squared error reduces rapidly. With the increase of training samples, decreasing speed of the mean squared error slows down and the mean squared error gradually approaches the optimal value. When the number of training samples reaches 7500, the mean squared error is almost consistent with the optimal value. That is, it has reached the convergence state.

**Figure 13:**
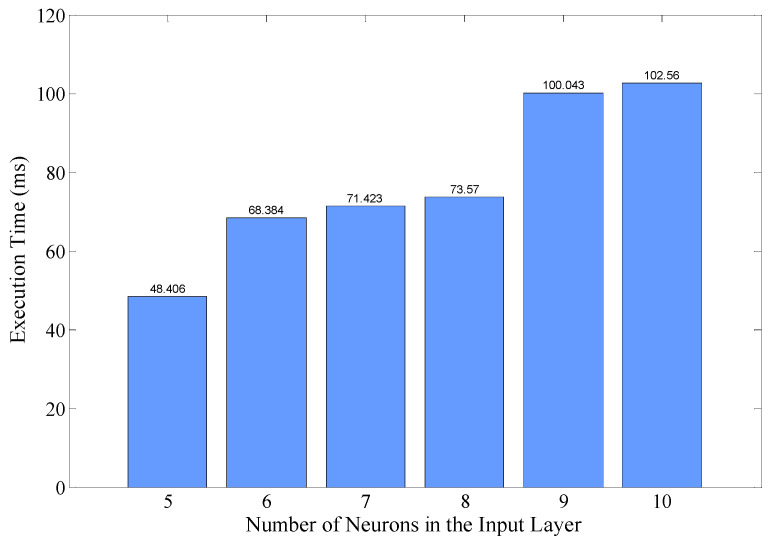
Execution overhead of the proposed method on TelosB.

The trained model was implemented on TelosB, a typical sensor node. Then, its execution time was evaluated carefully, as shown in Figure 13. As the power of TelosB is nearly constant when processing, the execution time is also an indirect representation of energy consumption. It is shown that with the model configuration described in Section 4, executing a PRR prediction takes about 102.56 ms. Considering the low-end microcontroller used in TelosB (16 bit MSP430 with 5 MHz highest clock frequency), this value is not surprising. However, as link quality prediction is not always running, this overhead is acceptable in practice. If more powerful microcontrollers such as ARM Cortex M0 could be utilized, the execution time is expected to be significantly reduced.

Execution time with different number of neurons in the input layer were also evaluated, also shown in Figure 13. It should be noted that when the number of neurons changes, the model should be retrained on desktop and deployed again on sensor nodes. As expected, the execution time reduces when the number of neurons decreases. However, with the number of neurons decreased, the accuracy of prediction will be affected inevitably, as less historical information could be utilized. Tradeoff should be made according to the specific needs of accuracy and execution time in practice.

## 7. Conclusions

Agile and accurate link quality prediction is essential for upper-layer protocols to select better links for communication, and thereby improving the network efficiency effectively. Predicting physical layer parameters first and then evaluating the link quality based on the mapping models between such parameters and PRRs has become one of the main methods for link quality prediction. However, existing methods ignore the temporal correlations of physical layer parameter series when predicting, which leads to large deviations between the predicted values and the actual ones. In addition, the impact of link fluctuations is not considered sufficiently, resulting in higher errors under moderate and sudden changed links with larger fluctuations.

In view of these problems, this paper proposes a more effective link quality prediction method RNN-LQI. RNN-LQI adopts RNN to predict the LQI series, and then evaluates the link quality according to the fitting model of LQI and PRR. On the one hand, this method could better mine the inner relationship among LQI series with the help of short-term memory capability of RNN, which helps to reduce the impacts of prediction errors. On the other hand, it could alleviate the impacts of link fluctuation on PRR mapping by taking advantage of the higher resolution of LQI in the transitional region, which helps to reduce the impacts of mapping errors.

To analyze the advantages of the proposed method, three methods of the same type were chosen for comparison. Compared with these methods, RNN-LQI shows higher prediction accuracy under different link qualities. Especially under moderate and sudden changed links with larger fluctuations, prediction errors of RNN-LQI reduce at least by 14.51% and 13.37%, respectively. It means that considering the temporal correlation of physical layer parameter series and the resolution of physical layer parameter mapping to PRR will help to realize more accurate link quality prediction.

The limitations of this method are mainly as follows: (1) Although the higher resolution of LQI in the transitional region has been utilized, the mapping error from physical layer parameters to PRR is only alleviated but not completely eliminated. (2) Offline test data collection and model training are needed to instantiate the proposed method, which makes it not adaptive to different environments and configurations. Therefore, the following future works may be feasible and valuable: (1) Link quality prediction methods with mapping error eliminated should be created to further improve the accuracy of prediction; (2) Online model training using the approach proposed in [15] could be considered and introduced to enhance the adaptability of the proposed method.

## Figures and Tables

**Table 1:** RMSEs of LQI prediction under different link qualities.

	Good Links	Moderate Links	Bad Links	Sudden Changed Links
RNN-LQI	***0.6015***	***8.8046***	***4.7920***	***5.8310***
WNN-LQI	0.7002	10.7078	5.2080	8.1484

**Table 2:** RMSEs of PRR prediction under different link qualities.

	Good	Moderate	Bad	Sudden Changed
RNN-LQI	0.0224	***0.2475***	***0.1661***	***0.1627***
WNN-LQI	0.0217	0.2895	0.1941	0.1878
RNN-SNR	***0.0076***	0.4403	0.2142	0.2247
WNN-SNR	***0.0076***	0.3964	0.1907	0.2747

## Data Availability

Not applicable.
